# Reversal of Roux-en-Y Gastric Bypass: A Multi-Centric Analysis of Indications, Techniques, and Surgical Outcomes

**DOI:** 10.1007/s11695-024-07650-2

**Published:** 2025-01-16

**Authors:** Liane Plath, Marie Vannijvel, Sietske Okkema, Ellen Deleus, Aaron Lloyd, Emanuele Lo Menzo, George Tadros, Ivana Raguz, Andres San Martin, Marko Kraljević, Styliani Mantziari, Sebastien Frey, Lisa Gensthaler, Henna Sammalkorpi, José Luis García Galocha, Vaishnavi Sujathan, Amalia Zapata, Talar Tatarian, Tom Wiggins, Ekhlas Samir Bardisi, Jean-Philippe Goreux, Yosuke Seki, Kazunori Kasama, Jacques Himpens, Marianne Hollyman, Richard Welbourn, Rajesh Aggarwal, Alec Beekley, Matias Sepulveda, Antonio Torres, Anne Juuti, Paulina Salminen, Gerhard Prager, Antonio Iannelli, Michel Suter, Ralph Peterli, Camilo Boza, Raul Rosenthal, Kelvin Higa, Matthias Lannoo, Eric Hazebroek, Christopher Pring, Will Hawkins, Guy Slater, Bruno Dillemans, Marco Bueter, Daniel Gero

**Affiliations:** 1 Department of Surgery, Männedorf Hospital, Männedorf, Switzerland; 2 Department of General Surgery, AZ Sint-Jan, Brugge, Belgium; 3 Department of Surgery, Rijnstate Hospital, Arnhem, Netherlands; 4 Department of General Surgery, Universitair Ziekenhuis Leuven, Louvain, Belgium; 5 Minimally Invasive and Bariatric Surgery, Fresno Heart and Surgical Hospital, Fresno, USA; 6 The Bariatric and Metabolic Institute, Cleveland Clinic Florida, Weston, USA; 7 Department of Surgery and Transplantation, University Hospital of Zurich, University of Zurich, Zurich, Switzerland; 8 Bariatric and Metabolic Center, Department of Surgery, Clínica Las Condes, Santiago, Chile; 9 Department of Visceral Surgery, Clarunis, Claraspital, Basel, Switzerland; 10 Department of Visceral Surgery, University Hospital of Lausanne, Lausanne, Switzerland; 11 Digestive Surgery and Liver Transplantation Unit, Centre Hospitalier Universitaire de Nice, Nice, France; 12 Department of Surgery, Medical University of Vienna, Vienna, Austria; 13 Department of Surgery, Turku University Hospital, Turku, Finland; 14 Department of Surgery, Hospital Clinico San Carlos, Complutense University of Madrid, Madrid, Spain; 15 Department of Upper Gastrointestinal and Bariatric Surgery, St Richard’s Hospital, University Hospitals Sussex NHS Foundation Trust, Chichester, UK; 16 Bariatric and Metabolic Surgery Center, Hospital Dipreca, Santiago, Chile; 17 Department of Surgery, Thomas Jefferson University Hospital, Philadelphia, USA; 18 Department of Upper Gastrointestinal and Bariatric Surgery, Musgrove Park Hospital, Taunton, UK; 19 Department of Surgery, Centre Hospitalier Universitaire de Saint-Pierre, Brussels, Belgium; 20 Department of Surgery, King Abdul Aziz University Hospital, Jeddah, Saudi Arabia; 21 Weight Loss and Metabolic Surgery Center, Yotsuya Medical Cube, Tokyo, Japan; 22 Department of Surgery, Hôpital Riviera-Chablais, Rennaz, Switzerland

**Keywords:** Roux-en-Y gastric bypass, Reversal, Conversion to normal anatomy, Indications, Outcomes, Recurrent weight gain, Secondary bariatric surgery, Complications, Malnutrition, Abdominal pain

## Abstract

**Background:**

Roux-en-Y gastric bypass may present long-term complications that require revisional surgery or even reversal to normal anatomy. Data on the indications, surgical technique, and outcomes of RYGB reversal remain scarce.

**Methods:**

We identified 48 cases of RYGB reversals with complete 90-day follow-up within a multi-centric international retrospective database of elective secondary bariatric surgery. The operations were performed between 2010 and 2024 in high-volume referral centers in Europe and USA. Data were collected on body weight, associated diseases, and on surgical outcomes up to 1-year postoperatively.

**Results:**

Patients were mainly female (81.3%) with a median age of 50 years (IQR 39–56). RYGB reversal was performed 7 years (median) after primary RYGB in patients with a BMI of 23.9 kg/m^2^ (IQR 20–27). Half of the patients underwent at least 1 bariatric revision before the reversal. Main indications for reversal were dumping syndrome (33.3%), excessive weight loss (29.2%), marginal ulcer (14.6%), malabsorption (12.5%), and abdominal pain (10.4%). Rate of conversion to open surgery was 8.3%, and the postoperative complications during the first year reached 50%, including 31.3% Clavien-Dindo grade I–II, 16.7% grade III–IV complications, and one death. At 1 year, the mean BMI of the cohort increased by 18% to 28.25 kg/m^2^; only 1 patient reached pre-RYGB BMI.

**Conclusion:**

Although RYGB is a theoretically reversible procedure, normal anatomy is re-established only in selected cases which are refractory to medical therapy and often also to revisional bariatric surgery. RYGB reversals entail high morbidity, while the extent of recurrent weight gain at 1-year post-reversal seems to allow patients to remain below the threshold of severe obesity.

## Introduction

Metabolic bariatric surgery (MBS) is an expanding field, with sleeve gastrectomy (SG) and Roux-en-Y gastric bypass (RYGB) being the most frequently performed procedures [1]. While primary MBS has low rates of early postoperative morbidity (i.e. bleeding or anastomotic/staple line leak), long-term complications may arise [2, 3]. After RYGB, these can include the so-called “dumping” syndrome [4], reactive hyperinsulinemia with hypoglycemia [5], excessive weight loss [6] or recurrent weight gain [7–9], marginal ulcer [10], suicidal behavior [5], excessive alcohol consumption [11], vitamin or mineral malabsorption resulting in anemia [12, 13], or chronic abdominal pain [14], which may pose a challenge for successful symptomatic control [15]. In cases that are refractory to conservative multidisciplinary management, surgical treatment classified as revisional, conversional, or reversal by re-establishment of the normal pre-bariatric anatomy may become necessary [14, 16].

**Fig. 1:**
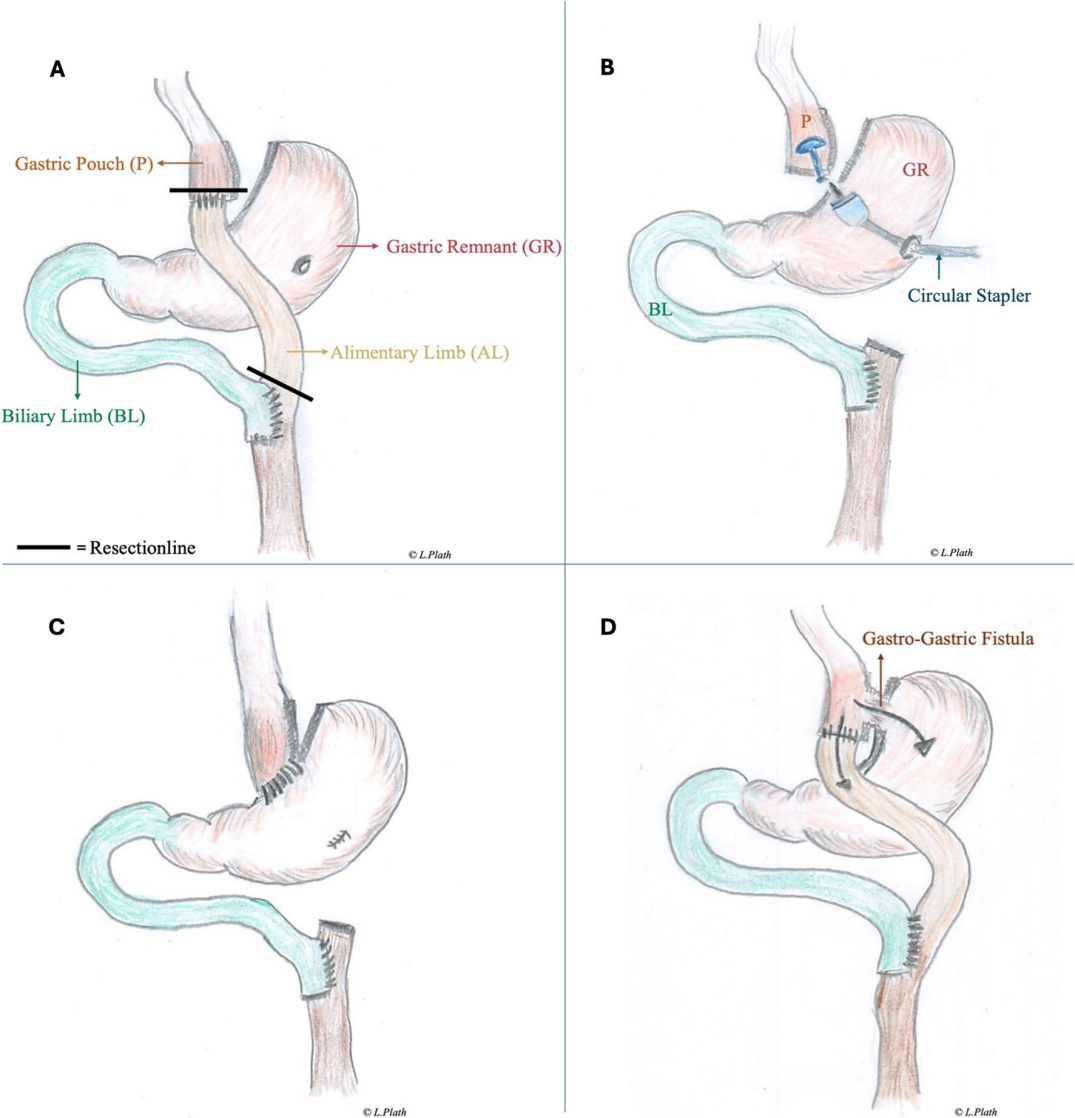
The captures **A**–**C** depict key steps of the surgical technique of laparoscopic reversal of Roux-en-Y gastric bypass (RYGB), while capture **D** shows an alternative approach. **A** Resection of the alimentary limb (optional step). **B** Gastro-gastric anastomosis using the circular stapler (linear stapler and hand-sewn anastomosis are also possible). **C** Final anatomy after reversal, with resection of the alimentary limb. **D** Functional reversal of the RYGB by creation of a gastro-gastric anastomosis

The reversal of RYGB is feasible laparoscopically [17]; however, indications and the surgical technique lack standardization. Different approaches have been reported in the literature, which include the following concepts: (1) reversal to normal anatomy with resection of the previous gastroenterostomy with linear stapler, resection of the Roux-limb and leaving the jejunojejunostomy intact, reconstruction with an anastomosis of the gastric pouch to the gastric remnant with circular or linear stapler, or hand-sewn [18] (Fig. 1A–C). (2) In case of short total intestinal length (<400 cm), re-anastomosis of the Roux-limb to the biliopancreatic limb [19] including checking the patency of the pylorus and in case of stenosis, performing a Heineke-Mikulicz pyloroplasty [20]. Lastly, (3) functional reversal with the creation of a gastro-gastric anastomosis for specific indications (i.e., dumping syndrome, malnutrition, fatigue, diarrhea); this technique encompasses shorter operation duration and potentially lower complication rates; nevertheless, it may increase the acidity exposure of the gastro-jejunostomy [21] (Fig. 1D).

Early postoperative morbidity is higher after secondary MBS than after primary MBS [22]. Consequently, perioperative aspects, such as timing, preoperative patient optimization, technical details of the reversal procedure, and centralization to high-volume centers, need to be considered [23]. One of the largest international databases on secondary MBS has been compiled by the Global Benchmarks in Secondary Bariatric Surgery Collaborative Group [22], which was used in the current study to provide a better understanding on the most common indications, perioperative management, and outcomes of patients who have undergone a reversal after RYGB in high-volume expert centers.

## Methods

### Data Origin

The current study is a multi-centric international retrospective in-depth analysis of prospectively collected clinical data on patients undergoing elective reversal of RYGB to pre-RYGB anatomy. We extracted all cases of reversal surgeries in patients with RYGB as primary MBS from the Global Benchmarks in Secondary Bariatric Surgery database of 5349 secondary MBS cases [22]. Additionally, 7 cases of RYGB reversals were included from the MBS referral center in Chichester, UK. The final database used to identify cases for this study was compiled by 19 expert centers from 4 continents which all met criteria of excellence, surgical volume and experience, infrastructure including intensive care unit, and a prospectively maintained database, as described previously [22]. The inclusion period spanned from 01/2010 to 02/2024 with a minimum mandatory follow-up of 90 days. Benchmark cases (i.e., lower risk) were defined using the following exclusion criteria: previous laparotomy, diabetes mellitus, sleep apnea, cardiopathy, renal insufficiency, inflammatory bowel disease, immunosuppression, thromboembolic events, BMI ≥50 kg/m^2^, or age ≥65 years. De-identified patient-specific data without any personal identifiers were transferred from each participating center to the core study team securely and were audited for integrity and completeness. In case of missing data, participating centers were requested to submit additional information based on the latest available follow-up.

### Outcomes of Interest

The outcomes of interest were related to surgical indications, timing between the RYGB and reversal, surgical safety and quality, reflected by the rate of short- and long-term complications. We also recorded patient demographics such as sex, age, body mass index (BMI) before 1st MBS, BMI before reversal surgery, and BMI 1 year postoperatively. Surgical morbidity was graded according to the Clavien-Dindo classification (CDC) [24], and to demonstrate cumulative morbidity, the Comprehensive Complication Index (CCI) was also applied. CCI reflects the burden of postoperative morbidity on a scale from 0 (no complication) to 100 (death) [25].

### Data Analysis

Descriptive statistics and data visualization were performed using Excel 365® (Microsoft Inc., Redmond, Washington, USA) and the R software version 4.2.3 (The R Foundation for Statistical Computing, Vienna, Austria). Discrete variables were described by count (percent), and continuous variables were described by median with interquartile ranges (IQR). Subgroups were compared using the “tableone” R package, which uses the ANOVA test to compare datasets with a normal distribution and the Kruskal-Wallis rank sum test for comparing datasets without a normal distribution.

## Results

### Patient Characteristics

We identified 48 cases of elective reversal of RYGB stemming from 9 different bariatric centers located in Europe and in the USA, which represented ~1% of their secondary MBS activity. Most patients were female (81.3%) with a median age of 50 years (IQR 39–56.5). The proportion of benchmark cases was 62 %. The median BMI at reversal was 23.9 kg/m^2^ (IQR 20.4–27.4). Half of the patients had already at least 1 bariatric revision before the reversal; the indications of previous revisions were not captured. Reversal was performed at a median of 2.7 years (IQR 0.8–6.8) after the last MBS and 7 years (IQR 3.2–13.9) after the RYGB. Major obesity-associated diseases at reversal surgery included arterial hypertension (35.4%), depression (33.3%), gastroesophageal reflux disease (GERD) (31.3%), dyslipidemia (22.9%), osteoarticular disease (20.8%), type 2 diabetes mellitus (14.6 %), cardiac arrhythmia (12.5%), sleep apnea (6.3%), and hyperuricemia (4.2%).

### Indication for Reversal of RYGB

**Fig. 2:**
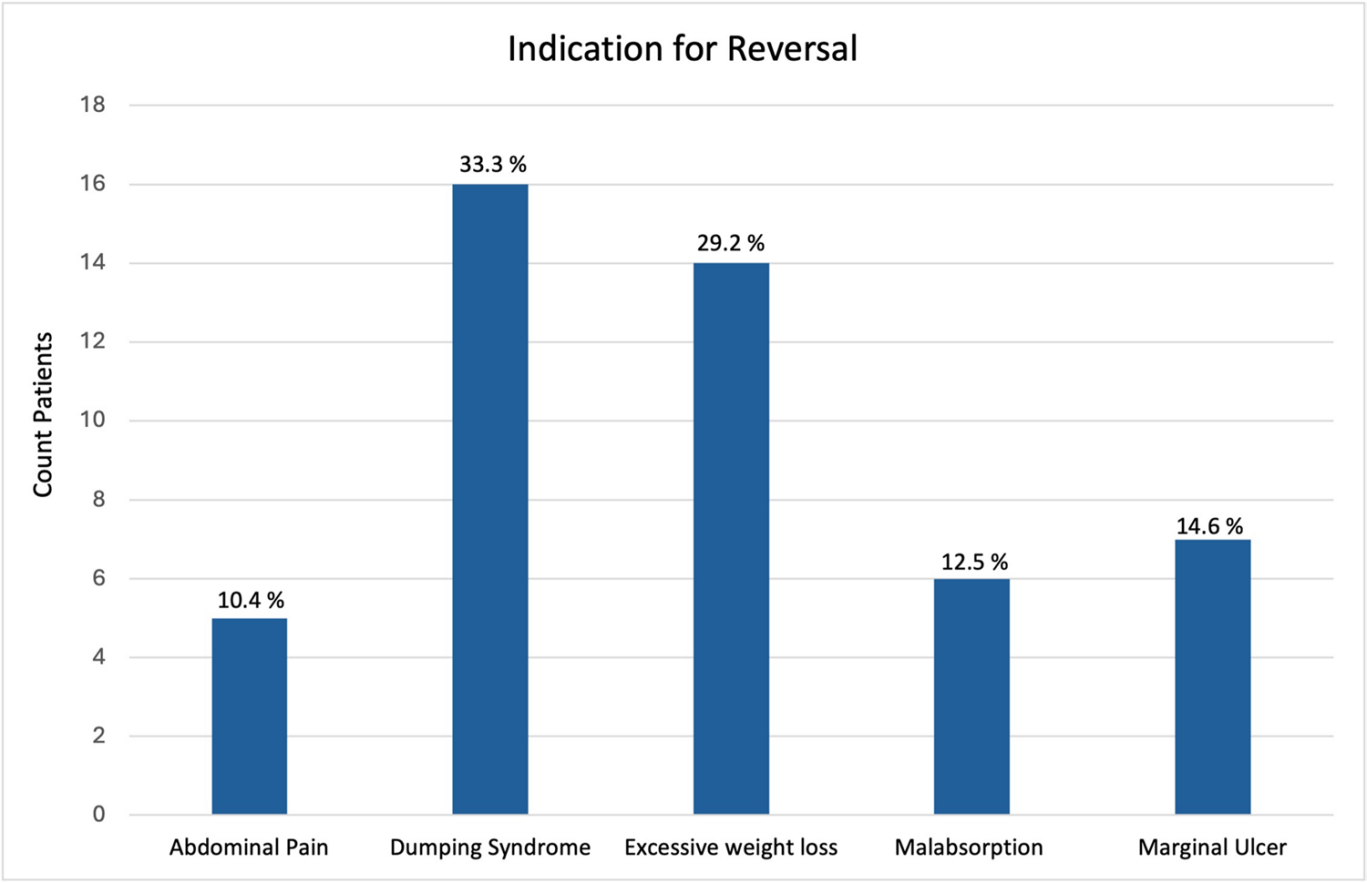
Distribution of the indications of elective reversal of Roux-en-Y gastric bypass

The main indications for reversal were dumping syndrome (33.3%) and excessive weight loss (29.2%), followed by marginal ulcer (14.6%), malabsorption (12.5%), and abdominal pain (10.4%) (Fig. 2). In patients undergoing reversal for excessive weight loss, the excess BMI loss was ≥100%, but no other standardized criteria were in place for patient selection.

### Surgical Technique

In all cases, a laparoscopic approach was used primarily with the need for conversion to open surgery in 4 cases (8.3%). All patients underwent a one-step procedure for the re-establishment of normal anatomy. The gastro-gastric anastomosis was done by stapler in 21 cases (43.8%), hand-sewn in 3 cases (6.3 %), and in 24 cases the technique of anastomosis could not be retrieved. The median operation duration was 107 min (IQR 82–141). Intraoperatively, a drain was placed in 25 cases (52.1%). Surgical teaching was performed, at least in part, in more than half of the cases (56.3%).

### Surgical Outcomes

The rate of follow-up at 90 days was 100%, which dropped to 80.7% at 1 year. During the first postoperative year, the overall postoperative morbidity was 50%, with 31.3% CDC grade I–II, 16.7% CDC grade III–IV, and one mortality on postoperative day (POD) 7 (out of hospital, reason unknown) (Table 1).

**Table 1:** Postoperative morbidity and mortality in the cohort of 48 patients undergoing elective reversal of Roux-en-Y gastric bypass to normal anatomy

Morbidity and mortality (*n*)	Until discharge	< 30 days postoperative	< 90 days postoperative	< 365 days postoperative	> 365 days postoperative (median follow-up: 1.5 years)	Overall
Ileus	2 (4.17%)	0	0	2 (4.17%)	0	4 (8.33%)
Anastomotic leak	4 (8.33%)	0	0	0	0	4 (8.33%)
Abdominal pain	1(2.08%)	0	0	1 (2.08%)	2 (4.17%)	4 (8.33%)
Bleeding	1 (2.08%)	0	0	0	0	1 (2.08%)
Wound infection	0	0	1 (2.08%)	0	0	1 (2.08%)
Pneumonia	1 (2.08%)	0	0	0	0	1 (2.08%)
Ulcer	0	0	0	0	2 (4.17%)	2 (4.17%)
Portal vein thrombosis	0	1 (2.08%)	0	0	0	1 (2.08%)
Dysphagia	0	0	1 (2.08%)	0	0	1 (2.08%)
Death	0	1 (2.08%)	0	0	1 (2.08%)	2 (4.17%)
CCI (mean ± SD)	5.67 ± 2.17	10.07 ± 20.92	13.03 ± 20.92	19.22 ± 33.73	22.39 ± 35.22	

*CCI*, Comprehensive Complication Index

Beyond 1 year, there was one non-procedure-related death (suicide at POD 411 in a patient with borderline personality disorder). Overall, 5 patients (10.4%) needed a re-operation after reversal (*n* = 3 gastro-intestinal leakage, *n* = 2 mechanical ileus), out of which three could be performed laparoscopically. Another patient with a gastrointestinal leak was treated medically and by keeping the prophylactically placed abdominal drain longer in situ without the need of any subsequent surgical intervention. Two patients presented with a postoperative ileus on day 3 and day 6 which resolved conservatively. One patient (2.1%) required ICU treatment postoperatively. The 4 cases of postoperative abdominal pain were reported by patients who did not undergo reversal due to pain as main indication for reversal. Hospital stay ranged from 1 to 34 days (median 5 days).

### Weight Trajectories Following Reversal of RYGB to Normal Anatomy

**Fig. 3:**
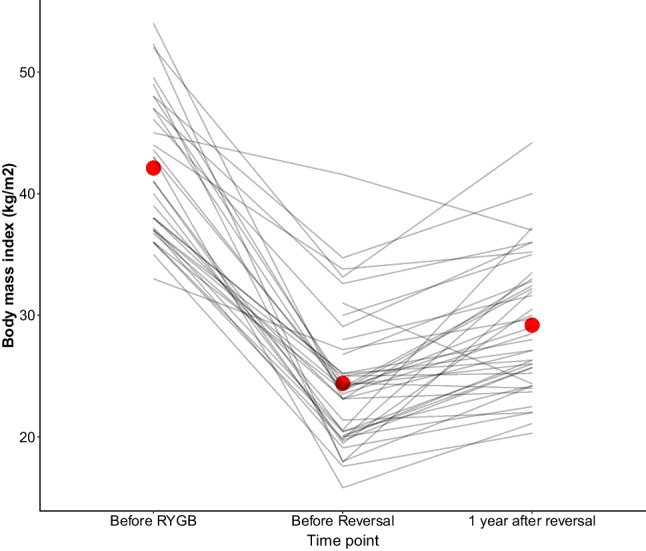
Body mass index trajectories in the cohort of 48 patients undergoing reversal of Roux-en-Y gastric bypass (RYGB) to normal anatomy. Red dots show groups means at different time points; grey lines connect the values of individual patients

The mean BMI of patients undergoing reversal did not reach the BMI threshold of severe obesity at 1-year postoperatively (Fig. 3). The overall weight gain represented an increase of 18% from the nadir BMI (from 23.9 to 28.2 kg/m^2^).

**Fig. 4:**
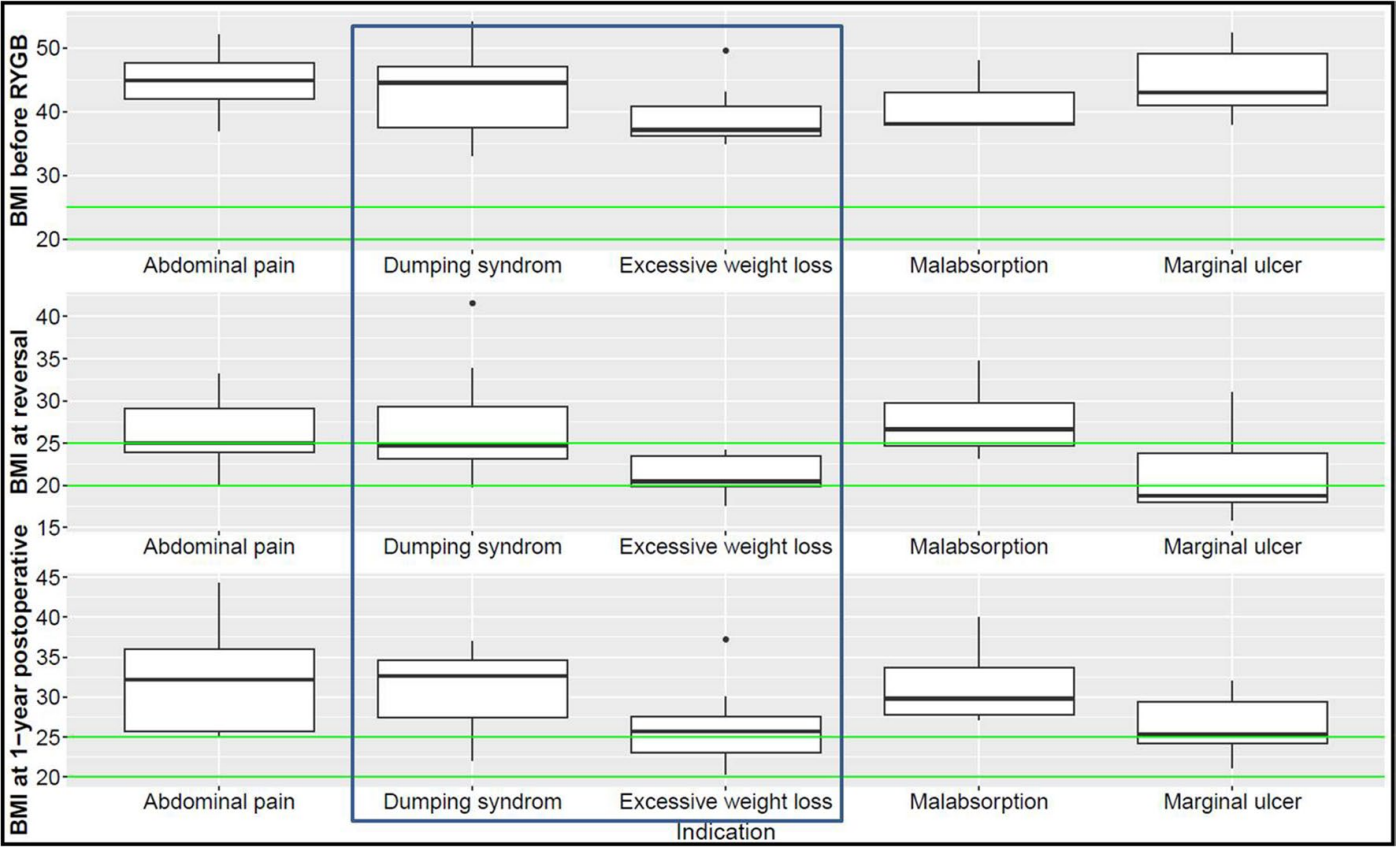
Body mass index (BMI) trajectories per subgroup based on the indication for reversal of Roux-en-Y gastric bypass to normal anatomy. Green lines indicate the healthy BMI range. The blue frame highlights the subgroups with *n* > 13

Subgroup analysis based on the indication for reversal identified differences in the post-reversal BMI trajectories (Fig. 4). The highest post-reversal weight gain was observed in patients who had abdominal pain, dumping syndrome, or marginal ulcer as main indication

## Discussion

This multicenter analysis provides valuable insight on the main indications and surgical outcomes of RYGB reversals. The main findings can be summarized as (1) reversal of RYGB to normal anatomy is a rarely performed MBS procedure (in the order of <0.1% of the MBS caseload), lacking standardized indications and surgical technique [26]; (2) the postoperative morbidity is by magnitudes higher than the morbidity of primary MBS (conversion to open surgery is >8% and serious adverse events affect ~20% of patients during the first postoperative year, including mortality); and (3) the post-reversal weight gain at 1 year is limited, and most patients do not reach the BMI threshold of severe obesity. The findings clearly advise that reversibility should not be used as a major argument to opt for RYGB over SG as primary MBS. In practice, the reversal is hardly ever done (we had to screen an international database of over 5300 secondary MBS surgeries to identify 48 elective cases), and in the rare cases when it is performed, most patients already had to experience other revisional bariatric surgeries and/or upper-gastrointestinal endoscopies before the indication is posed. Further, patients face a significantly higher perioperative risk, mainly due to the higher odds of postoperative sepsis and/or mechanical bowel obstruction, mandating reoperations, sometimes along with intensive care unit admission. And lastly, in the first postoperative year after reversal, we did not objectify a tendency of major weight recurrence; in most cases, the patients’ pre-bariatric BMI territory was not approached. Of note, reversal of RYGB seems to have its niche in the bariatric emergency setting as well, in case of infarcted alimentary limb for example.

Obesity is a chronic disease, and most patients require a durable intervention to maintain their body weight and obesity-associated disease profile in a relatively healthy range [27]. It has been observed that removal of gastric band without conversional MBS leads to recurrent weight gain, deterioration of physical and psychiatric associated diseases, and lower quality of life [28, 29]. More recently, it was also shown that cessation of treatment with glucagon‐like peptide‐1 receptor agonists (GLP‐1-RA) leads to recurrent weight gain [30]. Liraglutide has a rather short half‐life, with systemic concentration clearance of 2 days. Accordingly, the weight regain trajectories are driven by the duration of treatment and tolerated dosage and are likely to mirror the pace of weight loss when the drug was started [30]. Against this background, it is somewhat surprising that this study did not find rapid weight regain trajectories in patients undergoing reversal to normal anatomy after RYGB. The physiologic explanations are most likely manifold and may include early satiation mediated by the distention of the neo-gaster, as well as by the changes in absorption [31] and intestinal signaling due to the shorter total intestinal length [32, 33], whenever the reversal technique includes resection of the Roux-limb. Additionally, postbariatric adoption of a healthier lifestyle via behavioral changes in food intake and regular physical activity may protect against relapsing obesity [34, 35]. Post-reversal recurrent weight gain was the lowest in the subgroup of patients with malabsorption (^~^21%), whereas patients with dumping syndrome had a regain of up to 32% of their nadir BMI, supporting the theory that in some patients the postprandial nutrient sensing and consequent behavioral conditioning after RYGB becomes so potent that it prevents the intake of sufficient amount of calories [36]. The recurrent weight gain in patients with preoperative abdominal pain and marginal ulcer is most likely explained by the resolution of their food aversion conditioned by previous unpleasant postprandial experiences.

The indications for reversals of RYGB in this study do not mirror the trends that have been observed previously [19, 37]. A recent survey found that abdominal pain was the most frequent (93%) cause of reversal, even though it is often not alleviated surgically [38]. Based on the current study, abdominal pain represented only 10.4% of indications and no post-reversal pain-related reinterventions or readmissions have been documented in this subgroup. Of note, pain-related postoperative complications have been documented in 8.3% of the entire cohort. However, as postoperative pain was not assessed systematically, this condition might well be underreported. Another discrepancy was found for refractory marginal ulcers, which was reported to be the main indication for reversal (54%) by others [19, 37], while it only represented 14.6% of the indications in this study. Marginal ulcer is a frequent (>10%) complication of RYGB, most commonly caused by Helicobacter pylori infection [39], tobacco use, or non-steroidal anti-inflammatory drugs. All of these factors can be prevented or treated medically [40, 41]; however, gastroduodenal ulcers may even relapse after reversal of RYGB [19].

While the operation duration of RYGB reversal (median 107 min) is comparable to that of primary RYGB [3, 42], all other perioperative quality indicators are not in its favor. The rates of conversions to open surgery, admissions to ICU, reoperations, overall complications, and length of stay are fundamentally more prohibitive, even when surgeries are performed in high-volume centers of excellence. The reasons leading to this complication profile were not directly measured in this study, but are presumably related to the poorer ability of tissue healing due to malnutrition [4, 43, 44] and to intraoperative difficulties related to adhesions from previous operations. There is therefore a need for careful patient prehabilitation by a multidisciplinary team, and data support the beneficial effect of centralization of complex bariatric cases to regional MBS centers [45].

The main strength of this study is related to the granularity of the data on indications, surgical techniques, and outcomes captured in the Global Benchmarks in Secondary Bariatric Surgery Collaborative Group database, derived from selected high-volume bariatric centers of excellence from different continents. This approach allowed the assessment of 48 elective cases of RYGB reversals, with a complete 90-day and remarkably high 1-year follow-up rate. However, we acknowledge that despite the international collaborative effort, the cohort remains relatively small, which may limit the external generalizability of events with low frequency, such as postoperative mortality or ICU admissions. Another limitation is the lack of information on the rate of concomitant resection of the Roux limb during RYGB reversals and data on the total remaining intestinal length. Third, the database did not include systematic information on patients’ peri-operative nutritional status and self-reported quality of life or pain levels, which would have been an important addition to the understanding of the risks and benefits of RYGB reversals. Fourth, the functional success rate of the reversals was not directly assessed; therefore, future studies are needed to explore if patients’ expectations are met on the long term.

## Conclusion

In summary, reversal of RYGB is an infrequent procedure, with its main indications being dumping syndrome, excessive weight loss, marginal ulcer, malabsorption, or abdominal pain. Laparoscopic RYGB reversal is feasible; however, patients need to be informed of a moderately elevated rate of conversions to open surgery and a significantly higher rate of severe postoperative complications than in primary MBS. At 1-year postoperatively, most patients seem to remain below the BMI threshold of severe obesity, undermining the fear of imminent weight recurrence that is frequently observed after the removal of gastric bands or after the cessation of medical weight loss therapies.

## Data Availability

The data that support the findings of this study are not openly available due to reasons of sensitivity and are available from the corresponding author upon reasonable request. Data are located in controlled access data storage at the University Hospitals Zurich.

## Abbreviations

*BMI*: Body mass index
*RYGB*: Roux-en-Y gastric bypass
*MBS *: Metabolic bariatric surgery
*SG*: Sleeve gastrectomy
*POD*: Postoperative day
*CDC*: Clavien-Dindo classification
*CCI*: Comprehensive complication index
*SD*: Standard deviation
*GERD*: Gastroesophageal reflux disease
*ICU*: Intensive care unit
*GLP‐1-RA*: Glucagon‐like peptide‐1 receptor agonists
*d*: Days
*IQR*: Interquartile range
*n*: Count
*J-J*- *Anastomosis*: Jejunojejunostomy
*RL*: Roux limb
*AL*: Alimentary limb
*BL*: Biliary limb
*GR*: Gastric remnant
*ExWL*: Excessive weight loss
